# The use of respiratory muscle training in patients with pulmonary dysfunction, internal diseases or central nervous system disorders: a systematic review with meta-analysis

**DOI:** 10.1007/s11136-022-03133-y

**Published:** 2022-04-23

**Authors:** Luisa Cacciante, Andrea Turolla, Giorgia Pregnolato, Sara Federico, Francesca Baldan, Anna Rutkowska, Sebastian Rutkowski

**Affiliations:** 1 Laboratory of Rehabilitation Technologies, Hospital San Camillo IRCCS, Venice, Italy; 2 Department of Biomedical and Neuromotor Sciences, Alma Mater University of Bologna, via Massarenti, 9, Bologna, Italy; 3 Operative Unit of Occupational Medicine, IRCCS Policlinico Sant’Orsola-Malpighi, Bologna, Italy; 4 Faculty of Physical Education and Physiotherapy, Opole University of Technology, Opole, Poland

**Keywords:** Respiratory muscle training (RMT), Pulmonary dysfunction, Quality of life, Dyspnoea, Respiratory function

## Abstract

**Objective:**

The aim of this systematic review with meta-analysis was to evaluate the effectiveness of RMT in internal and central nervous system disorders, on pulmonary function, exercise capacity and quality of life.

**Methods:**

The inclusion criteria were (1) publications designed as Randomized Controlled Trial (RCT), with (2) participants being adults with pulmonary dysfunction caused by an internal disease or central nervous system disorder, (3) an intervention defined as RMT (either IMT or EMT) and (4) with the assessment of exercise capacity, respiratory function and quality of life. For the methodological quality assessment of risk of bias, likewise statistical analysis and meta-analysis the RevMan version 5.3 software and the Cochrane Risk of Bias Tool were used. Two authors independently analysed the following databases for relevant research articles: PubMed, Scopus, Cochrane Library, Web of Science, and Embase.

**Results:**

From a total of 2200 records, the systematic review includes 29 RCT with an overall sample size of 1155 patients. Results suggest that patients with internal and central nervous system disorders who underwent RMT had better quality of life and improved significantly their performance in exercise capacity and in respiratory function assessed with FVC and MIP when compared to control conditions (i.e. no intervention, sham training, placebo or conventional treatments).

**Conclusion:**

Respiratory muscle training seems to be more effective than control conditions (i.e. no intervention, sham training, placebo or conventional treatment), in patients with pulmonary dysfunction due to internal and central nervous system disorders, for quality of life, exercise capacity and respiratory function assessed with MIP and FVC, but not with FEV1.

## Introduction

Early identification of respiratory muscle weakness is important as a diagnostic and prognostic factor as well as for the implementation of appropriate therapeutic strategies [1]. Inspiratory muscle dysfunction due to obstructive pulmonary disease or neuromuscular disorders can result in reductions in inspiratory muscle strength and endurance, thus contributing to dyspnea, decreased exercise tolerance, and impaired quality of life [2]. Impairment of respiratory function after stroke related to dysphagia and ineffective cough may increase the risk of pneumonia, which has been reported as a leading cause of extravascular death after stroke [3]. In patients with respiratory disorders, muscle weakness can lead to dyspnea and hypercapnia which affects components of physical performance [4]. Impaired respiratory muscle strength and functional capacity are often reported in patients with lung or heart disease [5]. Breathing alterations are related to a decrease in physical activity and therefore, to a reduction in the ability to carry out the activities of daily life [6]. Thus, implementing interventions that have the potential to improve respiratory function and, consequently, to prevent morbidity and mortality for a chronically ill patients is vindicated [7].

Respiratory muscle training (RMT) has long been used in many medical disciplines [8]. Respiratory muscles respond to training similarly to any other skeletal muscle [9]. In this regard, RMT consists of repetitive breathing exercises with hand-held respiratory trainer devices to provide pressure threshold or flow-dependent resistance against inhalation (inspiratory muscle training [IMT]) and/or exhalation (expiratory muscle training [EMT]) [10] to stimulate this musculature and to produce changes in the muscles’ structure. The diversity of potential applications for IMT makes it impossible to provide specific guidance for all potential applications. However, there are major classes of diseases or conditions where studies support a beneficial effect of RMT on clinical outcomes in subgroups of patients: cardiorespiratory, neuromuscular, surgical, healthy aging. The misconception remains that only patients with evidence of inspiratory muscle weakness or ventilatory limitation can benefit from IMT. However, even when inspiratory muscle weakness is not an inclusion criterion, improvements in dyspnea and exercise capacity still occur after IMT [11]. To date, no reports of adverse events after IMT have been found, but there is a theoretical risk of barotrauma-related events. However, in patients with coronary artery disease, it is reasonable to minimize hypocapnia by using a slower respiratory rate. In patients who have experienced an acute exacerbation or chest infection, clinical judgment should be used regarding the risk of inducing hyperfatigue of inspiratory muscles. In this situation, it may be appropriate to reduce the intensity and/or frequency of IMT use. The vast majority of patients undergoing RMT are those with long-term chronic conditions such as respiratory disease, heart failure, obesity, and neuromuscular disease. For these patients, the timing of IMT will depend on disease management policies. RMT can be provided in the patient's home as part of primary care provided by a specialist nurse or physiotherapist or in an outpatient setting. Evidence suggests that RMT can be used both as a stand-alone intervention and as part of a multidimensional rehabilitation program in a wide range of patients. Furthermore, comorbidities that preclude physical training are not a barrier to RMT, thus making it an ideal intervention for patients with severe impairment [12].

However, despite the fact that certain effects of RMT have been shown in previous research on several populations (e.g. stroke survivors [13, 14], respiratory [11, 15–17], cardiac [18, 19], or cancer patients [20]), evidence regarding the efficacy of respiratory muscle training is still inconclusive and controversial. RMT reduce the occurrence of respiratory complications in stroke survivors immediately or even 3–12 months after treatment initiation [21], however evidence regarding change in swallowing function after stroke remains lacking [22]. Inspiratory muscle training benefits were demonstrated on quality of life, dyspnea, and exercise capacity in patients with pulmonary dysfunctions [4]. Regarding cardiac patients it has been shown that inspiratory muscle training improves diaphragmatic mobility in patients following cardiac surgery [23], likewise improve pulmonary function, such as FVC [24]. Little is known about clinical efficacy of RMT in cancer patients. Conflicting results have been reported regarding increases in respiratory muscle strength after lung cancer surgery and oxygenation [25]. Although, IMT and aerobic exercise improves muscle strength and exercise capacity in lung cancer patients [26]. Furthermore, the assessment of training efficacy from most work is often limited to measures of lung function and/or physical performance. Therefore, we decided to perform a systematic review along with a meta-analysis on the effectiveness of respiratory muscle training in internal and central nervous system disorders, evaluating the combined effect on respiratory function, exercise capacity and quality of life.

## Methods

The study design was set as a systematic review and meta-analysis and was conducted according to the PRISMA 2020 guidelines [27]. The protocol was prospective registered in the PROSPERO database (registration number: CRD42020216639) on 07.12.2020.

### Electronic searches

Publications were searched in PubMed, Embase, Web of Science, Scopus, and the Cochrane Library. The last search was launched on 8th February 2021. A detailed description of the search strategy is presented in the supplementary materials (see Appendix 1).

### Study selection

In this review, we included (1) publications designed as Randomized Controlled Trial (RCT), with (2) hospitalized adults patients (i.e. > 18 years) with pulmonary dysfunction caused by an internal disease or central nervous system disorder, (3) an intervention defined as RMT (either IMT or EMT) compared to all other treatments or no intervention, and (4) with the assessment of exercise capacity, respiratory function and quality of life as primary outcomes. The inclusion criteria of device type and training characteristics were not considered in order to include the largest number of studies. A similar approach was applied to the choice of intervention location (inpatient, home-based, outpatient). The review included publications in English, Italian and Polish. Grey literature was not searched in this review. Conversely, we excluded non-RCTs and studies involving healthy adults, children or participants with sleep disorders. Furthermore, we excluded studies in which all the three outcomes (i.e. exercise capacity, respiratory function and quality of life) were not assessed. For study selection through abstract screening, two reviewers, independently, screened studies that were identified through the electronic search engines already mentioned, based on title and abstract, using the free tool Rayyan (https://rayyan.qcri.org/). A third reviewer was selected to solve any disagreements. At the end of this process, full text of the articles were obtained, and the same procedures were used for full text screening and for the assessment of the methodological quality (risk of bias assessment).

### Outcomes

We aimed to assess three main outcomes, i.e. respiratory function, exercise capacity and quality of life. We assessed respiratory function with parameters of respiratory muscle weakness (i.e. FEV1, FVC, MIP) and exercise capacity with the 6-min walking test (6MWT) or other scales that aim to assess exercise capacity. Finally, we wanted to evaluate the improvement of perceived-quality of life by using questionnaires that assess changes in the perception of quality of life (e.g. St. George respiratory questionnaire, SF-36).

### Data extraction and management

Screening of research records was conducted with the procedure already mentioned, then a full-text screening was conducted with the same procedure. A data extraction form was filled with all the relevant data, i.e.: authors and year of publication, number of participants and their characteristics, type of interventions and training, outcome measures and conclusions drawn by authors.

### Assessment of risk of bias in included studies

Studies included in the review underwent a methodological quality assessment for risk of bias using the Cochrane Risk of Bias Tool [28]. We evaluated the following domains: (1) Selection bias: random sequence generation; (2) Selection bias: allocation concealment; (3) Performance bias: blinding of participants and personnel (4) Detection bias: blinding of outcome assessment; (5) Attrition bias: incomplete outcome data; and (6) reporting bias: selective reporting. We coded risk of bias for each domain as “high risk”, in case of a high possibility in the occurrence of bias; “low risk”, in case of a low possibility of bias; “unclear risk”, when we could not exactly define the real incidence of bias. Finally, potential publication bias was explored by visual inspection of funnel plots.

### Data synthesis and statistical analysis

We used Review Manager 5.3 to conduct review, to assess the methodological quality of trials through the risk of bias tables, and for statistical analysis. We attempted to categorize the included interventions along three outcomes group: (1) respiratory function, (2) exercise capacity and (3) quality of life. Treatment effects were evaluated using Mean Difference (MD) for homogeneous outcome measures or Standardized Mean Difference (SMD) for the outcomes evaluated with different scales. Confidence Interval (CI) for continuous outcomes was identified at 95%. Statistical heterogeneity was assessed with the I^2^ statistic. We deemed to perform the analyses with 95% CI, based on random effects model, as we assumed that there would be the presence of heterogeneity due to the different population included in the review and the different types of training provided [29]. We planned a subgroup analysis in relation to medical diagnosis (i.e. internal diseases, pulmonary diseases, stroke and leukemia). In the case of no data available for synthesis, an email was sent to the corresponding author. We assumed a 2-week waiting period for a response.

## Results

**Fig. 1:**
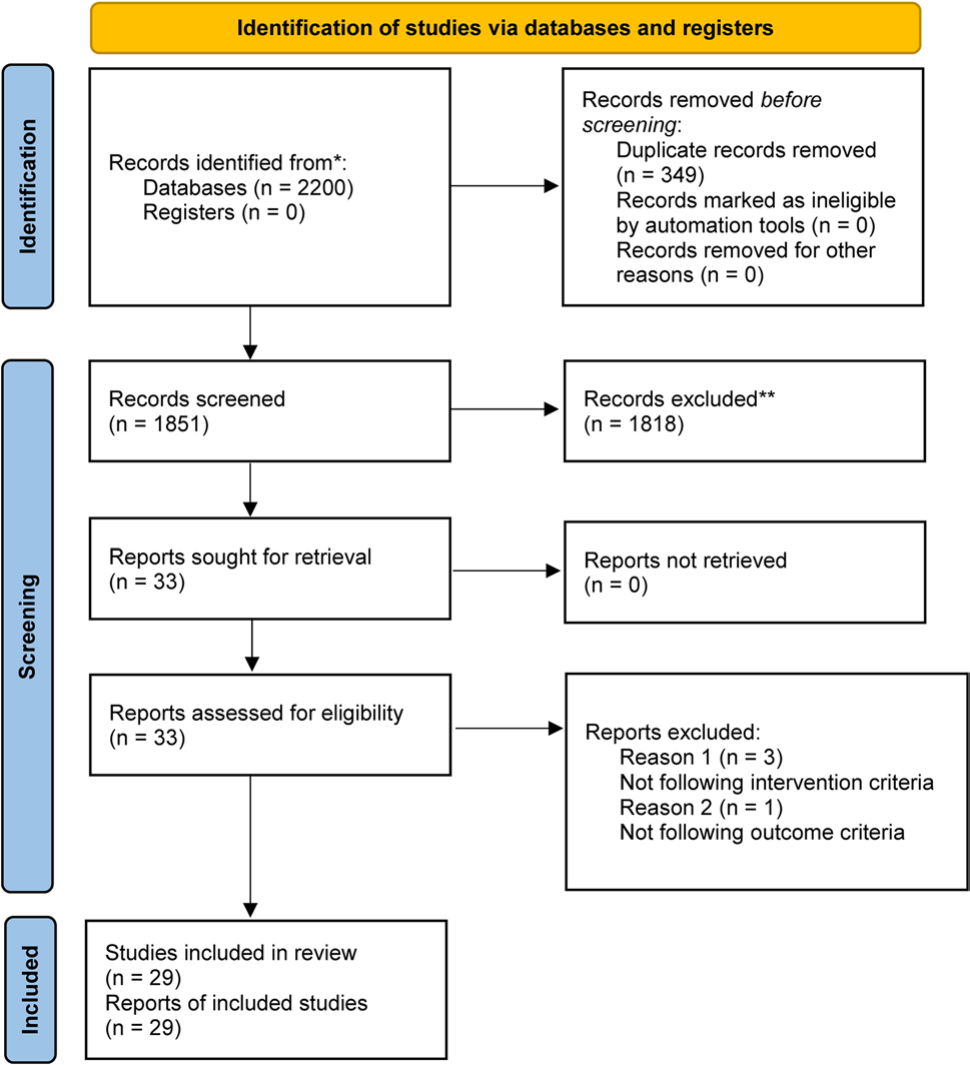
Flow diagram for the study selection process

Our search identified 2220 results from 5 electronic databases. The following numbers were retrieved from publications in each of the databases: PubMed: 166; Scopus: 1596; Cochrane Library: 145; Web of Science: 44, and Embase: 269. After removing 349 duplicates, 1851 abstracts remained for screening. We excluded 1818 records with unrelated target topics and then assessed for eligibility a total of 33 full text articles. The main reasons for rejecting abstracts were: different study design than specified in the inclusion criterion and different study population. After full-text screening, 29 studies met the inclusion criteria for qualitative analysis. At the end of the process, 25 studies remained for quantitative analysis, as 4 studies did not report data as Mean and Standard Deviations. The PRISMA flowchart of the review process is shown in Fig. 1.

### Characteristics of included studies

All the included studies were RCTs focusing on the use of RMT for pulmonary dysfunction. Among the 29 included studies, 8 included participants with internal diseases [30–37], 19 studies focused on the treatment of patients with pulmonary diseases [2, 38–55] and the remaining 2 studies included patients with stroke and leukemia, respectively [56, 57]. The overall number of participants included within trials was 1155, with 619 patients involved in RMT programs and 536 patients treated in control groups.

With regard to the intervention provided, all the included studies used a threshold-loading device. Only one study investigated the effects of EMT on lung function, exercise tolerance, symptoms and health-related quality of life in severe COPD patients [52], whereas 5 studies evaluated the effectiveness of RMT for patients with Restrictive Thoracic Disease [41], stroke [56], leukemia [57] and COPD [47, 48]. The remaining 23 studies provided IMT programs, in order to evaluate their efficacy, in patients with COPD [2, 38–40, 42, 44, 45, 50, 51, 53, 54], asthma [43], lung cancer [46], bronchiectasis [49], in patients with chronic airflow limitation [55], heart failure [31, 33, 34, 37], in patients who underwent coronary artery bypass graft surgery [32, 36] and Heart Valve Replacement Surgery [30], and in patients with pulmonary arterial hypertension [35].

Three studies compared the experimental intervention to placebo intervention [30, 31, 53], whereas eight studies used sham training as comparator [35, 40, 41, 47, 50, 52, 54, 55]. In 4 studies the experimental intervention was compared to no intervention [42, 44, 45, 48] and 12 studies compared the use of RMT to conventional rehabilitation [2, 32, 33, 36–39, 43, 46, 49, 56, 57]. Sadek et al. tested the combination of high-intensity aerobic interval training and IMT, and compared it to IMT alone, high-intensity aerobic interval training alone and no intervention [34]. Finally, Majewska-Pulsakowska et al. compared the combination of IMT and cycle ergometer to IMT alone, cycle ergometer alone and no treatment [51].

The dose of therapy varies between studies, ranging from a total of 20 sessions over 10 days of treatment to a maximum of 288 sessions over 1 year.

A detailed description of the characteristics of included studies is presented in supplementary material (Table 1).

**Table 1:** Characteristics of the selected studies

Author, Ref. (country)	Groups characteristic	*n*	Interventions	Outcome measures	Training type	Conclusions
*Internal diseases*						
Cargnin et al. [30] (Brazil)	(1) Patients with HVRS + IMT-G (EG) (2) Patients with HVRS + IMT-PG (CG)	(1) 13 (2) 12	The groups were treated equally, according to routines of the post-operative heart surgery unit at the hospital. The patients were followed daily during the hospital stay. Prior to hospital discharge, patients received a training orientation and a diary in which to record the home training sessions. After discharge, individuals returned weekly to the hospital to measure inspiratory muscle strength. On the same day, a training load adjustment was made for the IMT-G	Spirometry, MIP, 6MWT, SF-36	Three days after HVRS, participants received a pressure linear load device for IMT. The device was to be used to perform 30 ventilatory cycles, twice/day, 7 days/week, for 4 weeks. During training, the participants were instructed to maintain diaphragmatic breathing. For subjects in IMT-G, the inspiratory load was adjusted to 30% of MIP. This was adjusted weekly so that the relative load was kept at 30% of MIP during the IMT period	IMT increased inspiratory muscle strength and lung function values, both returning to the pre-operative level, and improved the functional capacity to levels above the pre-operative in patients undergoing HVRS
Dall’Ago et al. [31] (Brazil)	(1) Patients with CHF + IMT (EG) (2) Patients with CHF + P-IMT (CG)	(1) 16 (2) 16	Patients received either IMT or P-IMT for 30 min 7 times per week, for 12 weeks using the Threshold Inspiratory Muscle Training device. The P-IMT followed the training schedule with no respiratory load	FVC, FEV-1, Pthmax/PImax, 6MWT, Minnesota Living With Heart Failure Questionnaire, Pthmax, VO2peak, VE peak, R peak	During training, patients were instructed to maintain diaphragmatic breathing, with a breathing rate at 15 to 20 breaths/min. For the IMT group, inspiratory load was set at 30% of maximal static inspiratory pressure, and weekly training loads were adjusted to maintain 30% of the PImax	A short-term, home-based program of IMT results in marked improvement in inspiratory muscle strength and endurance, as well as in quality of life, in CHF patients with inspiratory muscle weakness
Miozzo et al. [32] (Brazil)	(1) Patients who underwent CABG + AE + IMT (EG) (2) Patients who underwent CABG + AE (CG)	(1) 13 (2) 11	Patients allocated to the AE + IMT performed a protocol of high-intensity IMT followed by an aerobic exercise protocol, for 12 weeks. Patients allocated to the CG performed the same aerobic exercise protocol performed in the EG, during the same 12 weeks. These patients did not perform the high-intensity IMT protocol	6MWD, PeakVO_2_, MIP, MEP, SRT, SF-36	The high-intensity IMT protocol was performed with a linear pressure loading device. IMT was performed for 12 weeks and the protocol consisted of five sets with 10 repetitions each until the 8th week and progression of the number of sets and repetitions from the 8th to 12th week	There was an improvement of all outcomes in both groups, but high-intensity IMT was not able to provide additional benefit in most of the outcomes, being observed only in inspiratory muscle strength
Palau et al. [33] (Spain)	(1) Patients with HFpEF + IMT + standard care (EG) (2) Patients with HFpEF + standard care alone (CG)	(1) 14 (2) 12	Patients in the EG group performed an IMT using a threshold inspiratory muscle trainer, and were instructed by a therapist and educated to maintain diaphragmatic breathing during the training period. Patients in the CG were checked weekly by a respiratory therapist who measured their MIP each time	6MWT, peak VO_2_, VO_2_ at anaerobic threshold, VE/VCO_2_ slope, MIP, Minnesota Living With Heart Failure Questionnaire	Patients allocated to the IMT arm were instructed to train at home twice a day, for 20 min during 12 weeks using a threshold inspiratory muscle trainer. The subjects started breathing at a resistance equal to 25–30% of their MIP for 1 week. The resistance was then modified each session according to the 25–30% of their MIP measured	A 12- week home-based IMT intervention in HFpEF patients with reduced aerobic capacity and preserved inspiratory muscle strength was associated with significant and clinical relevant improvement in exercise capacity and QoL
Sadek et al. [34] (Lebanon)	(1): CHF patients + HI-AIT (2): CHF patients + IMT (3): CHF patients + combined training ( HI-AIT & IMT) (4): CHF patients + no rehabilitation	(1) 13 (2) 12 (3) 12 (4) 13	-Control Group: no treatment -AIT Group: aerobic training -IMT Group: 20 min of inspiratory muscle training using Power Breathe device -AIT&IMT Group: combined two modalities (aerobic training followed by inspiratory training, with 5 min rest between)	-Pulmonary function test: FEV1, FVC -6MWT -Quality of life: MLWHF -Respiratory function: MIP, MEP, SMIP, MET	3 sessions/week, for 12 weeks AIT training: 4 × 4 min at 60% to 90% of MHR, 5 × 2 min at 50% of the maximum intensity of the workload IMT training: 20 min of training using Power Breathe device. The device strengthened the breathing muscles (diaphragm and intercostals) The breathing rate: 15 to 20 breaths per minute The training load was set at 60% of MIP	The combination of the HI-AIT and the IMT resulted in additional benefits in respiratory muscle function, exercise performance, and quality of life compared to that of HI-AIT or IMT alone IMT was better than control n all outcome
Saglam et al. [35] (Turkey)	(1) PAH patients + IMT (2) PAH patients + sham IMT protocol	(1) 14 (2) 15	-Group 1 (Training group): IMT program -Group 2 (Control group): sham IMT program	-Functional capacity: 6MWT -Respiratory muscle strength: MIP, MEP, peak expiratory flow -Pulmonary function: FEV1, FVC -Quality of life: NHP -Fatigue: FSS -Dyspnea perception: MMRC dyspnea scale	Patients were trained using an inspiratory threshold-loading device. 30 min/day, 7 days/week, for 6 weeks The Training Group received IMT at 30% of MIP. The MIP was adjusted weekly The Control Group received sham IMT at a fixed workload of 10% of MIP	IMT promoted significant improvements in: -Respiratory muscle strength -Functional capacity IMT training reduced: -Dyspnea during activities of daily living -Fatigue
Savci et al. [36] (Turkey)	(1) CABG patients + usual care + IMT (2) CABG patient + usual care	(1) 25 (2) 25	-Intervention Group = usual care + IMT -Control Group = usual care	-Functional capacity: 6MWT -Respiratory muscle strength: MIP, MEP (cmH_2_O) -Pulmonary function: FEV1, FVC, FEV 1/FVC -Quality of life: NHP -Anxiety and Depression: HADS	The Control Group: usual care (mobilization, active exercises of upper and lower limbs, chest physiotherapy) The Intervention group: in addiction to usual care, had IMT under the supervision of a physical therapist For 30 min, 2times/day, for 10 days They used a threshold-loading device: from 15 to 45%	IMT results in faster recovery of inspiratory muscle strength, functional capacity, intensive care unit stay, quality of life and psychosocial status after CABG
Winkelmann et al. [37] (Brazil)	(1) Patients with CHF and IMW + AE + IMT (2) Patients with CHF and IMW + AE	(1) 12 (2) 12	-AE Group: aerobic exercise training -AE + IMT Group: aerobic exercise training + IMT	-Functional capacity: 6MWT -Respiratory muscle strength: PImax, PEmax -Pulmonary function: VO_2_peak, VCO_2_peak, V_e_peak, OUES -Quality of life: MLWHF	The IMT inspiratory exercise by using Threshold equipment for 30 min, 7 times per week, with an inspiratory load at 30% of PImax Aerobic training: consists of cycle exercise (3 sessions/week) at a cadence of 60 rpm. During the first 2 weeks, the duration of the exercise is 20 min, and 5 min are added every 2 weeks, until the exercise reaches 45 min Both treatments last 12 weeks	The addition of IMT to AE results in improvement in cardiorespiratory responses to exercise in selected patients with CHF and IMW The 6MWT and MLWHF scores also improved similarly after both training programs
*Pulmonary diseases*						
Ahmad et al. [2] (Malaysia)	(1) Individuals with pulmonary dysfunction COPD + IMT (EG) (2) Individuals with pulmonary dysfunction COPD + chest physiotherapy treatment (CG)	(1) 9 (2) 9	Both groups performed the training procedures for 4 weeks EG received: chest physiotherapy, active cycle breathing technique (ACBT) and postural drainage (PD) and Inspiratory Muscle Training (IMT) for 15 min, once a day, 5 days a week for 4 weeks CG: only chest physiotherapy treatment	FEV1, FEV1/FVC, PImax, 6MWT, SGRQ,	IMT for 15 min, once a day, 5 days a week for 4 weeks IMT protocol was performed using a “patient module of pressure threshold Respifit-S unit” for 30 repetitions in 15 min with intensity of 30% of baseline maximum inspiratory pressure (PImax). IMT was progressed until 60% of subjects PImax with 10% resistance increment weekly	Combination of IMT with chest physiotherapy treatments for at least 4-week duration can produce benefits for lung function, inspiratory muscle strength, exercise tolerance and quality of life among COPD patient
Arnedillo et al. [38] (Spain)	(1) Individuals with pulmonary dysfunction COPD + IMT (EG 1) (2) Individuals with pulmonary dysfunction COPD + oronosal breathing (ONB) (EG 2) (3) Individuals with pulmonary dysfunction COPD + standard medical recommendations (CG)	(1) 7 (2) 5 (3) 4	Patients from EG 1 nad EG 2 carried out a supervised RP for 8 weeks, 3 days per week. The pulmonary RP included therapeutic education and training sessions lasting 60 min with a warming up phase, a main phase and a recovery phase. The training program included aerobic exercise on cycle ergometers and on treadmills, strengthening of lower and upper limb muscle groups, breathing exercises (pursed lip breathing, diaphragmatic and abdominal breathing and diaphragmatic mobility) and stretching exercises. EG 1 with added IMT	mMRC, CAT, PImax, 6MWT,	In the EG 1 group, for restricted nasal breathing, at the beginning of the training program, the small size device was used (4 mm). The size of the device was progressively increased according to the patient adaptation to the 5 or 6 mm device, depending on the score on Borg’s perceived exertion scale. If the patient had a score under 4 after the RP sessions, the size of the FB device was increased. The device was used during the RP and patients were encouraged to do physiological breathing by nasal inspirations and mouth expirations	IMT provides greater improvements in quality of life, dyspnea, exercise capacity and inspiratory muscle strength compared to patients that did not use it
Beaumont et al. [39] (France)	(1) Individuals with pulmonary dysfunction COPD + PR + IMT (EG) (2) Individuals with pulmonary dysfunction COPD + PR (CG)	(1) 74 (2) 75	The pulmonary rehabilitation programme for EG and CG group was conducted over 4 weeks, 5 days per week and included aerobic exercise on a cycle ergometer and a treadmill, strengthening of lower and upper limb muscle groups, a therapeutic educational programme, aerobic gymnastics in groups, a smoking cessation programme and sociopsychological and dietary advice	MDP, 6MWT, mMRC, SGRQ, PImax, FEV1, FEV1/VC, TLC	In the EG group, all subjects trained their inspiratory muscles daily during two sessions of 15 min each, five times a week, over 4 weeks. The patients had to breathe slowly with an increased tidal volume; after 10 inspirations, they could have a break by breathing at rest for a short time. The cycle of 10 inspirations was repeated 15 times. The inspiratory muscle training was performed using a threshold inspiratory muscle trainer (PowerBreathe) at a resistance generating a pressure corresponding to 50% of the initial PImax for each session. The intensity was increased (+ 10%) after 10 days of training during the programme to reach 60% of the initial PImax	Pulmonary rehabilitation with IMT was not found to be superior to pulmonary rehabilitation without IMT in terms of dyspnoea, quality of life or exercise capacity (6MWD) improvement, despite a significantly higher improvement of PImax in the IMT group
Beckerman et al. [40] (Israel)	(1) Individuals with pulmonary dysfunction COPD + IMT (EG) (2) Individuals with pulmonary dysfunction COPD + training with very low load (CG)	(1) 21 (2) 21	All subjects trained daily in two sessions of 15 min each, six times a week for 12 months The training was performed for 1 month under the supervision of a respiratory therapist followed by home training, verified by a respiratory therapist daily by phone and once weekly by a personal visit, for the next 11 months The control group trained for the same sessions with a fixed load that required generation of mouth pressure of 7 cm H2O	6MWT, PImax, POD, SGRQ, FVC, FEV1	The training was performed using a threshold inspiratory muscle trainer (POWERbreathe). The subjects started breathing at a resistance that required generation of 15% of Pimax for 1 week. The load was then increased incrementally, 5 to 10% each session, to reach generation of 60% of Pimax at the end of the first month. IMT was then continued at 60% of the Pimax adjusted monthly to the new Pimax achieved	The study shows that during IMT in patients with COPD, there is an increase in exercise capacity, improvement in quality of life, and decrease in dyspnea
Budweiser et al. [41] (Germany)	(1) Patients with restrictive thoracic disorders + RMT (EG) (2) Patients with restrictive thoracic disorders + sham training (CG)	(1) 15 (2) 15	Patients in both groups were instructed to perform the training procedures for 3 months. Patients were encouraged to only slowly increase their daily training time. The target was to achieve a duration of at least 10 min twice a day within the second week, or until discomfort occurred	IVC, TLC, Pimax, Pemax, 12-s MVV, PaO_2_, PaCO_2_, Maximal work rate, VE, VO_2_ peak, 6MWD, Time of NPPV, Severe Respiratory Insufficiency questionnaire	Isocapnic hyperpnea training: a portable RMT appliance was used, consisting of a hand-held unit with tubing, connected to a rebreathing bag (50% of IVC). Subjects must fill and empty the rebreathing bag completely during their breathing maneuver. A sideport in the middle of the connecting piece contains a hole and a valve that ensures inhalation of additional fresh air during inspiration and allows breathing partly out during expiration; thus, hyperventilation is prevented	There is some evidence that in severe restrictive thoracic disorders, RMT performed as isocapnic hyperpnea over 3 months can improve respiratory muscle function
Chuang et al. [42] (Taiwan)	(1) Individuals with pulmonary disfunction COPD + IMT (EG) (2) Individuals with pulmonary disfunction COPD + no interventions (CG)	(1) 27 (2) 28	ET group was provided medical treatment and routine care, along with five sessions of threshold inspiratory muscle training per week (21–30 min per session), accompanied by a progressive increase in the pressure threshold over a period of 8 weeks. The CG was provided medical treatment and routine care only, without intervention	PImax, BDI, 6MWT, SF-36	The intervention was performed 8 weeks, with 5 sessions a week, lasted for 21–30 min, consisted of a cycle of 2-min inspiratory training with a pressure threshold loading device and 1-min of rest and then repeated s7 cycles. IMT intervention started with pressure of 15 cm H2O, after a week the pressure threshold loading was concurrently increased to 20 cmH2O. A midterm test was administered in the fourth week, and the pressure threshold loading was increased to 30 cmH2O. In the sixth week, the pressure was increased to 40 cmH2O	The study shows that significantly increases inspiratory muscle strength and endurance, improves outcomes of exercise capacity and both physiological and psychological components in the quality of life and decreases dyspnoea in patients with COPD
Duruturk et al. [43] (Turkey)	(1) Patients with asthma + IMT (EG) (2) Patients with asthma + education session (CG)	(1) 20 (2) 18	At the beginning of the study, all patients were provided with 30 min of education session on bronchial hygiene techniques and breathing training. Subjects in the control group received only the education session, subjects in the IMT group made additional hospital visits, 3 times per week for 6 weeks for breathing training and the IMT program using a patient-specific threshold pressure device (POWERbreathe)	FVC, FEV-1, FEV-1/FVC, MIP, MEP, 6MWT, FSS, SGRQ, London Chest Activity of Daily Living scale	The POWERbreathe was applied for 30 dynamic inspiratory efforts, twice daily, for 6 weeks, at a pressure threshold load that was 50% of MIP	Inspiratory muscle strength training in patients with asthma can lead to improvements in respiratory muscle performance, exercise capacity, activities of daily living, health-related quality of life, and a further decline in dyspnea and fatigue
Elmorsi et al. [44] (Egypt)	(1) Individuals with pulmonary disfunction COPD + muscle training + IMT (EG 1) (2) Individuals with pulmonary disfunction COPD + muscle training (EG 2) (3) Individuals with pulmonary disfunction COPD + no training	(1) 20 (2) 20 (3) 20	The program was conducted following a schedule of 24 visits in the 8 week period. Patients in group EG 1 and group EG 2 were subjected to a combination of lower limb endurance training using treadmill walking, upperlimb endurance training with a combination of arm raiseand arms together, upper limb strength training using handweights for biceps and triceps, and lower limb strengthtraining using straight leg raise	PImax, Pemax, 6MWT, SGRQ, mMRC, BODE, FEV1, FVC, FEV1/FVC	All subjects in group EG 1 were trained six times a week; each session consisted of 30 min, for 2 months using a Threshold inspiratory muscle trainer. Patients started breathing at a resistance that required the generation of 30% oftheir Pimax for one week. The load was then increased incre-mentally, 5–10%, to reach a generation of 60% of their PImax at the end of the first month. Specific IMT was then continued at 60% of their Pimax adjusted weekly to the new PImax achieved	MT plus peripheral muscle exercise training led to a significant improvement in PImax,PEmax,and 6-min walking distance (6MWD) compared to peripheral muscle exercise training alone. Both IMT plus peripheral muscle exercise training and peripheral muscle exercise training alone improved dyspnea, BODE index and SGRQ-C without significant differences between 2 groups For PImax,PEmax,and 6MWD; IMT provides additional benefits to peripheral mus-cle exercise training in COPD patients
Garcia et al. [45] (Spain)	(1) Individuals with pulmonary disfunction COPD + muscle training + IMT (EG) (2) Individuals with pulmonary disfunction COPD + no training (CG)	(1) 8 (2) 5	EG IMT programme The CG did not undergo any kind of training, being solely advised to continue with habitual daily activities	FEV1, FVC, MIP, MEP, SGRQ, MDI, ESWT	All patients in the EG group was allocated to a specific IMT programme with a Threshold device and using MIP resistance of between 40 and 50%. IMT duration was 30 min daily, five time weekly for a period of five consecutive weeks. Training was supervised by a physiotherapist and undertaken with control of breathing rhythm (six cycles per minute)	The use of IMT in patients with COPD induced an improvement in inspiratory muscle force with a consequent improvement in the quality of life in relation to symptoms
Huang et al. [46] (China)	(1) Patients with LC + IMT combined with CRT (combined PR group) (2) Patients with LC + conventional PR (single IMT group) (3) Patients with LC + routine preoperative preparation (CG)	(1) 30 (2) 30 (3) 30	PR program was primarily a physical-based intervention that focused on exercise endurance and resistance training or a combination of methods, such as IMT, and CRT, coupled with psychological educational guidance. Patients in the single IMT group received conventional single-mode IMT, and the CG patients underwent routine preoperative preparations, including preoperative education for in-hospital, preoperative preparation and essential encouragement or psychological caring	Clavien-Dindo Complication Classification System, 6-MWD, fatigue score, dyspnea score, PEF, FEV-1, FVC, DLCO, QoL evaluation	The patients in the combined PR group were treated for one week with high-intensity preoperative PR using IMT and aerobic endurance exercise. IMT involved abdominal and thoracic breathing training. This training used a respiratory training device. Patients performed these exercises for 20 min at least four times daily. Meanwhile, for CRT, a NuStep cross-training apparatus was used at a rehabilitation training center. Patients used the NuStep twice daily for 20 min per session	Compared with IMT program, by applying exercise regimens and increasing physical activity in LC patients with risk factors of PPCs, the combined program could better improve the exercise capacity, inspiratory muscle strength and QoL, which additionally contributed to alleviate the PPCs severity
Koppers et al. [47] (the Netherlands)	(1) Individuals with pulmonary dysfunction COPD + ERMT (EG) (2) Individuals with pulmonary dysfunction COPD + sham training (CG)	(1) 18 (2) 18	Endurance Respiratory Muscle Training was used in EG—but there is lack of informations about device and resistance used during training	FEV1, IVC, Pimax, Pemax, 6MWT, CRQ	RMET was performed by means of tube breathing. Maximum ventilatory capacity that can be sustained for 15 min is approximately 60% of maximum voluntary ventilation (MVV). Patients wore a nose clip and were instructed to take deep breaths Sham training was performed by breathing 6–7 times per minute through an incentive flowmeter, airflow resistance was set at 5% Pimax	Home-based RMET by means of tube breathing leads to a significant improvement of endurance exercise capacity, a reduction in perception of dyspnea, and an improvement in quality of life in patients with moderate-to-severe COPD
Leelarungrayu et al. [48] (Thailand)	(1) Individuals with pulmonary dysfunction COPD + standard RMT device (EG 1) (2) Individuals with pulmonary dysfunction COPD + prototype RMT device (EG 2) (3) Individuals with pulmonary dysfunction COPD + no training (CG)	(1) 10 (2) 10 (3) 10	6 week RMT training in EG 1 and EG 2 group In CG no interventions The patients received medication in the form of either a long-acting inhaled bronchodilator or a long-acting inhaled steroid,	PImax, Pemax, 6MWT, CCQ, Borg scale, FVC, FEV1, FEV1/FVC	The standard EG 1 or simple prototype EG 2 RMT device, with plastic caps having different sized holes (6, 4, and 2 mm diameter), was selected for both the training groups, which started inspiration through a 6 mm hole once daily for the first 2 weeks, before changing to 4 mm and 2 mm holes in the second and fourth week, respectively. Thirty slowly repeated inspirations passed through the device, with a 3-min interval of rest in each of four training sessions; thus, 20–30 min of RMT was completed The subjects initiated each inspiratory effort from residual volume and stroke in order to maximize the inspiratory volume. Therefore, 120 inspirations in a device with 3 resting intervals were performed in both the standard and simple prototype RMT groups	This simple prototype device can be used clinically in COPD patients as a standard device to train respiratory muscles, improving lung function and QOL RMT improves respiratory muscle strength and reduces dyspnea in COPD patients. Overall results in this study show that changes of FVC, FEV1/FVC, PImax, PEmax, QOL, and dyspnea score are similar between training with a simple prototype device and a standard RMT device
Liaw et al. [49] (Taiwan)	(1) Patients with bronchiectasis + IMT (EG) (2) Patients with bronchiectasis with any training program (CG)	(1) 19 (2) 19	The IMT program was started at an intensity of 30% MIP, and increased each week. A pressure threshold device was used. Patients in the control group did not receive any training programme. Both groups were monitored by telephone call once or twice a week until the end of the study	spirometry, SpO2, lowest SpO2, Borg Scale during 6MWT, 6MWD, 6Mwork, MIP, MEP and SGRQ	During training patients used a threshold device with an intensity of 30% MIP, which increased each week. Patients were asked to perform IMT for 30 min/day, at least 5 days/week, for 8 weeks. If the training sessions could not be completed due to increased resistance, the last part of the session was performed with the previous resistance setting	home-based pressure threshold resistive IMT did increase both inspiratory and expiratory respiratory muscle strength, but had no significant effect on respiratory function, quality of life and even walking capacity between groups in patients with bronchiectasis
Magadle et al. [50] (Israel)	(1) Individuals with pulmonary dysfunction COPD + GER + IMT (EG) (2) Individuals with pulmonary dysfunction COPD + GER + sham IMT training (CG)	(1) 16 (2) 15	The PR program was divided into the two phases: pre- and post-randomization. In the pre-randomization phase all patients participated in a general exercise reconditioning (GER) program that included lower extremity endurance exercise (walking or cycling), upper extremity exercise and strength training with free weights. This phase included 36 sessions of 1,5 h duration (three times a week for 12 weeks) After the first 12 weeks, half of the group was assigned to receive GER plus IMT (EG) using a pressure threshold device POWERbreathe The other half of the group undertook GER plus IMT sham training (CG). During this phase, GER took place for 1 h three times a week, for 6 months	FVC, FEV1, 6MWT, PImax, SGRQ, POD	There is a lack of protocol of IMT training	The addition of IMT did not yield further improvements in 6MWT, it did result in significantly greater improvements in quality of life and POD than GER alone
Majewska-Pulsakowska et al. [51] (Poland)	(1): COPD patients + IMT (2): COPD patients + CET (3): COPD patients + IMT + CET (4): COPD + no rehabilitation programs (CG)	(1) 8 (2) 9 (3) 13 (4) 13	-Group1: inspiratory muscles training -Group2: cycle ergometer -Group3: cycle ergometer + inspiratory muscle training -Group4: no treatment	-Pulmonary function: PI_max_, MET, FEV1 (L), FEV1 (% predicted) -Health-related quality of life: SGRQ -Tolerance to exercise: 6MWT	-Cycle ergometer: 8 weeks of intervention, for 3t/week. Each training session began (warm-up) and finished (relaxation) with a pedaling at a load of 10 W for 3 min -IMT: 8 weeks, 5 times a week, twice a day for 5–15 min. Performed by themselves at home on a Threshold IMT	The results demonstrate a significant improvement in the quality of life measured for Group 3 (IMT + cycle ergometer) in comparison with Group 4 (no treatment)
Mota et al. [52] (Spain)	(1) EG: COPD patients + Expiratory training group (2) CG: COPD patients + sham training group	(1) 10 (2) 8	-Exercise group: expiratory training with specific threshold (50% of MEP) -Sham group: expiratory training with no specific threshold	-lung function: MRC, FEV1, MEP; V_E_, V_t_ (other outcome about inspiratory capacity, with unclear abbreviation) -Exercise tolerance: 6MWT, Borg scale, VO_2 max_ -QoL: SGRQ	5 weeks, 3t/week, 30 min per session Patient had to breathe through an expiratory threshold device. Repeated cycles of 3 min of work, followed by 2 min of rest -Expiratory muscle training: the load was about 50% of MEP -Sham training: no load addiction to the threshold device	-Lung function remained unchanged after training -Exercise capacity, symptoms and quality of life significantly improved in Exercise Group -The improvement in both walking distance and the SGRQ score significantly correlated with changes in MEP
Riera et al. [53] (Spain)	(1) EG: COPD patients + IMT training (2) CG: COPD patients + no training	(1) 10 (2) 10	Inspiratory muscle training performed at home, with a incentive flowmeter device with visual feedback	-Pulmunary function: FEV1, FVC -Inspiratory Muscle Endurance Test (SIPmax) -PImax -Maximal exercise Capacity: VO_2_max; Wmax, V_E_ -Exercise performance: SWT -Dyspnea: BDI, TDI -QoL: CRQ	-1st part: 4 weeks, pre-training -2^nd^ part: 8 weeks, training phase -GroupT was trained with load 60 to 70% of the maximal sustained inspiratory pressure (SIPmax) -GroupC was trained at zero load (the flowmeter air leak was closed)	Targeted IMT improved dyspnea, increased the capacity to walk, and Improved quality of life in COPD patients
Schultz et al. [54] (Germany)	(1): COPD patient + highly intensive IMT (2): COPD patient + sham IMT	(1) 300 (2) 302	-Group 1 (intervention group) = usual care + highly intensive strength IMT -Group 2 (control group) = usual care + sham IMT	-Functional capacity: 6MWT -Respiratory muscle strength: PImax -Pulmonary function: FEV1, FIV1, FVC -Quality of life: SGRQ, CAT, CCQ -Dyspnoea: BDI, TDI	All patients receive rehabilitation programme for 3 weeks (i.e. endurance training, strength training, patient education, respiratory exercises, saline inhalation, …) The intervention group: IMT training is provided by using a threshold training device. The initial training load was 30% of PImax and was progressively increased to 60%. 7times/week, for 21 min The Control Group: patients use the same device, but with any valve. No threshold is used. 3times/week	IMT as an add-on to a 3-week pulmonary rehabilitation improves inspiratory muscle strength (PImax), but does not provide additional benefits in terms of exercise capacity, quality of life or dyspnoea
Seròn et al. [55] (Chile)	(1): CAL patients + IMT (EG) (2): CAL patients + SHAM (CG)	(1) 17 (2) 18	-EG = IMT with personalised load, based on PImax -CG = IMT with fixed load	-Respiratory muscle strength: PImax -Functional capacity: 6MWT -Pulmonary function: FEV1 -Quality of life: CRQ	Muscle strength is training Threshold Inspiratory Muscle Trainer device The threshold for the treatment group was Individualized: a load that is 40% of inspiratory muscle strength measured by PImax at baseline The Control Group works with the minimal loading offered by the exercise device The training are performed in a series of 5 repetitions with 1-min periods between them. From 10 to 30 min a day, for 3 weeks	Use of a threshold loading device is effective for strengthening inspiratory muscles as measured by PImax after the first month of training in patients with CAL The long-term effectiveness of such training and its impact on quality of life should be studied in a larger number of patients
*Stroke*						
Sutbeyaz et al. [56] (Turkey)	(1) STROKE patients + conventional rehabilitation + IMT (2) STROKE patients + conventional rehabilitation + BRT (3) STROKE patients + conventional rehabilitation	(1) 15 (2) 15 (3) 15	-Group 1: IMT Group. IMT + conventional rehabilitation -Group 2: BRT Group. Breathing retraining + conventional rehabilitation -Group 3: Control Group. Conventional rehabilitation	-Pulmonary function: FEV1, FVC, FEV1/FVC, VC, FEF, PEF, MVV, MIP, MEP -Ambulation status: FAC -Activities of daily living: Barthel Index -Respiratory muscle strength: PImax, PEmax -Quality of life: SF-36 -Dyspnoea	BRT training consists of: 15 min of diaphragmatic breathing, combined with pursed-lips breathing, and 5 min of air-shifting techniques, and 10 min of voluntary isocapnoeic hyperpnoea; it is performed daily for 6 weeks IMT training consists of: breathing exercises using a threshold inspiratory muscle trainer, with load of 40% PImax. Exercise intensity is gradually increased, 5–10% each session, to 60% of PImax as tolerated; it is performed in 2 session of 15 min each, 6 days/week, for 6 weeks Conventional rehabilitation is 5 days/week, for 6 weeks	A 6-week programme of IMT improves inspiratory muscle function in stroke patients. This was associated with an increase in lung volumes and improvements in exercise capacity, sensation of dyspnoea score and SF-36 domains
*Leukemia*						
El-Nahas et al. [57] (Egypt)	(1) Patients with Leukemia + chemotherapy and IMT (EG) (1) Patients with Leukemia + chemotherapy only (CG)	(1) 30 (2) 10	Patients who were included in the study group received IMT for 4 successive weeks, 5 sessions/week; patients who formed the control group, just received chemotherapy	MVV, 2MWT, FACT-G	Intervention in the EG lasted for 20 min: patients started breathing at a resistance of 30% of MIP, and then incrementally increased, based on patients’ perception of exertion. Then, the load was increased rapidly over the first 7 days up to 60–80% of the baseline MIP	The adjunctive physical therapy modalities with its various effects has been recommended, which can help in supporting the maintenance of cancer patients’ health-related quality of life

*EG* experimental group, *CG* control group, *COPD* Chronic Obstructive Pulmonary Disease, *IMT* inspiratory muscle training, *FEV1* Forced Expiratory Volume in the 1st second, *FEV1/FVC* Tiffeneau-Pinelli index, *6MWT* Six-minute walk test, *SGRQ* St. George’s Respiratory Questionnaire, *ACBT* active cycle breathing technique, *PD* postural drainage, *ONB* Oronosal breathing, *mMRC* modified Medical Research Council, *CAT* COPD Assessment Test, *MDP* Multidimensional Dyspnoea Profile, *FEV1/VC* forced expiratory volume in one second% of vital capacity, Tiffeneau index, *TLC* Total Lung Capacity, *POD* Perception of dyspnoea, *BDI* Baseline Dyspnoea Index, *PEmax* expiratory mouth pressure, *MIP* Maximal Inspiratory Pressure, *MEP* Maximal Expiratory Pressure, *ESWT* Endurance Shuttle walk Test, *MDI* The *Mahler’s Dyspnoea* Index, *CCQ* Clinical COPD Questionnaire, *GER* general exercise reconditioning, *GOLD* Global Initiative for Chronic Obstructive Lung Disease, *PAH* pulmonary arterial hypertension, *CABG* coronary artery bypass graft, *CAL* Chronic Airflow Limitation, *IMT* inspiratory muscle training, *6MWT* 6 min walking test, *PImax* maximal inspiratory pressure, *MET* metabolic equivalent, *FEV1 (L)* forced expiratory volume in one second, *QoL* quality of life, *VO*_*2 max*_ maximal oxygen consumption, *V*_*E*_ minute ventilation, *V*_*t*_ tidal volume, *CRQ* chronic respiratory questionnaire, *TDI* transitional dyspnea index, *SWT* symptom-limited test, *SIPmax* maximal sustained inspiratory pressure, *Wmax* maximal workload, *AIT* aerobic interval training, *MHR* maximal hearth rate, *FVC* forced vital capacity, *SMIP* sustained maximal inspiratory pressure, *MLWHF* Minnesota Living with Heart Failure Questionnaire, *FSS* Fatigue Severity Scale, *NHP* Nottingham Health Profile, *HADS* Hospital Anxiety and Depression Scale, *FIV1* forced inspiratory volume in one second, *IMW* inspiratory muscles weakness, *VC* vital capacity, *FEF* forced expiratory flow rate, *PEF* peak expiratory flow rate, *MVV* maximum voluntary: ventilation, *BRT* breathing retraining, *FAC* functional Ambulation Categories, *SF-36* The Medical Outcomes Study Short Form 36, *CHF* chronic heart failure, *OUES* oxygen uptake efficiency slope, *IVC* inspiratory vital capacity, *12-s MVV* 12-s maximal voluntary ventilation, *PaO*_*2*_ pressure of oxygen, *PaCO*_*2*_ pressure of carbon dioxide, *VO*_*2*_* peak* peak oxygen consumption, *6MWD* 6-min walking distance, *NPPV* non-invasive positive-pressure ventilation, *HVRS* heart valve replacement surgery, *IMT-G* inspiratory muscle training group, *IMT-PG* inspiratory muscle training placebo group, *P-IMT* placebo inspiratory muscle training, *Pthmax* maximal inspiratory pressure sustained for 1 min during incremental test, *R peak* peak respiratory exchange ratio, *CT* combined training, *LC* lung cancer, *CRT* conventional resistance training, *PR* pulmonary rehabilitation, *DLCO* diffusion capacity for carbon monoxide of the lung, *PPCs* post-operative pulmonary complications, *6Mwork* 6-min walking work, *AE* aerobic exercise, *SRT* sitting rising test, *HFpEF* Heart failure with preserved ejection fraction, *VCO*_*2*_ carbon dioxide production

### Excluded studies

We deemed 4 studies to be excluded, as in two studies no resistance training was provided in the experimental group [58, 59] and in one study the minimum load of the device (9 cmH2O) was kept constant during the study period in the control group [60]. One more study was excluded because the outcome quality of life was not investigated [61].

### Risk of bias in included studies

**Fig. 2:**
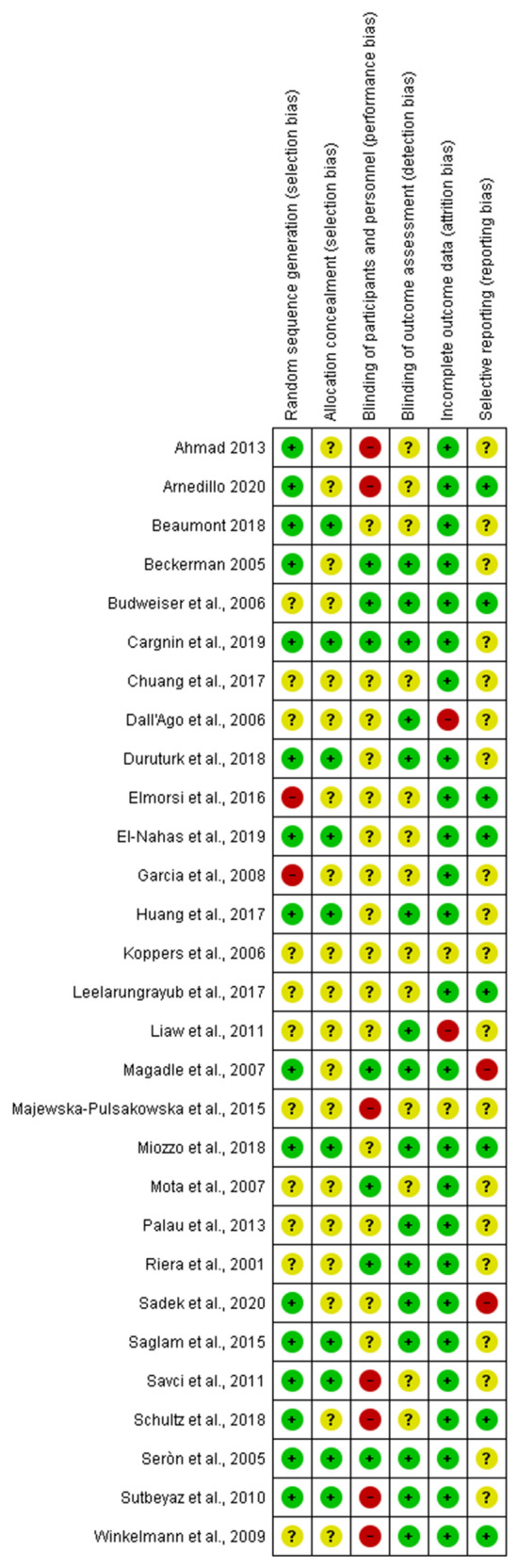
Summary of the risk of bias analysis

Figure 2 shows the risk of bias in the included studies.

- *Random sequence generation (selection bias)*: Sixteen studies were assessed with a low risk of bias, as the authors described a random component in the sequence-generation process, whereas two studies were judged with a high risk of bias, as randomization procedures were not appropriate. The remaining 11 studies were judged with an unclear risk of bias, as no information was provided.
- *Allocation concealment (selection bias)*: Ten studies had a low risk of bias in this domain, as the allocation methods used were appropriate. In 19 studies there was no information about allocation concealment procedures, resulting in an unclear risk of bias.
- *Blinding of participants and personnel (performance bias)*: Seven studies clearly stated that participants and therapists were blinded to intervention group allocation, whereas in other 7 studies no blinding was provided. It was unclear whether participants and therapists were blinded to intervention group allocation in 15 studies.
- *Blinding of outcome assessment (detection bias)*: In 16 studies the outcome assessor was unaware of the participants’ assigned interventions, so the risk of bias was low. In 13 studies the risk was unclear due to lack of information.
- *Incomplete outcome data (attrition bias)*: Twenty-five studies were assessed with a low risk of bias for this domain, as no missing data were found. Only one study had an unclear risk of bias because information was not reported and potential missing data were not provided. Outcome data were incomplete in the remaining 2 studies.
- *Selective reporting (reporting bias)*: In 8 studies the risk of bias was low, whereas it was unclear whether selective reporting occurred in 19 studies, as the study protocols were not available. In 2 studies selective reporting was identified.

### Effects of intervention

#### Comparison 1 quality of life

**Fig. 3:**
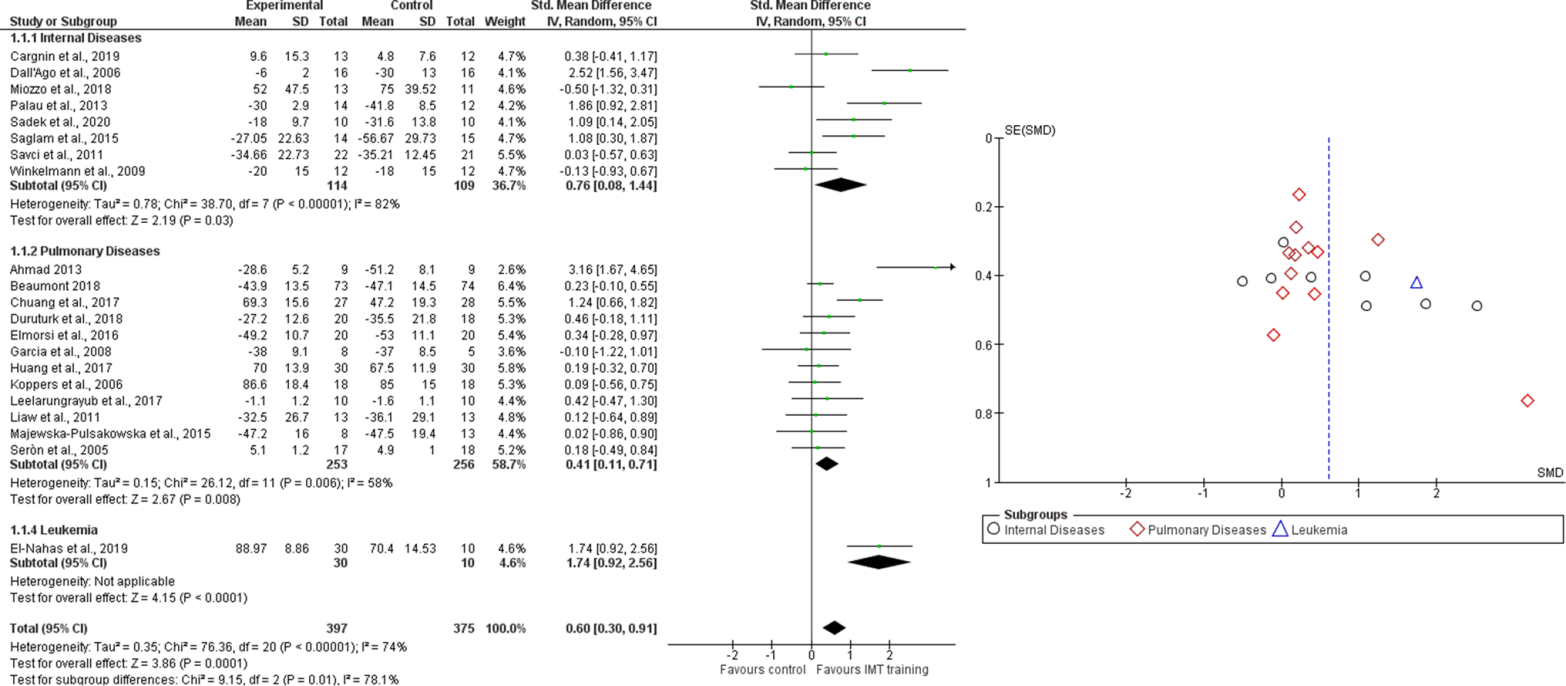
Comparison 1. RMT versus other interventions. Outcome: 1.1 Quality of Life, likewise Funnel plot of Comparison 1. RMT versus other interventions. Outcome: 1.1 Quality of Life

Twenty-one studies were analyzed for quality of life, with a total of 772 participants. Subgroup analyses were performed, based on medical diagnosis (i.e. internal diseases, pulmonary diseases and leukemia). The analyses were performed using Standardized Mean Difference (SMD) with random effects model, since all the included studies used different outcome measures for the same outcome and we expected high values of heterogeneity. The total result showed a significant difference in favour of RMT (SMD = 0.60, 95% CI 0.30 to 0.91, I^2^ = 74%), as well as for all the subgroups, i.e. internal diseases (SMD = 0.76, 95% CI 0.08 to 1.44, I^2^ = 82%), pulmonary diseases (SMD = 0.41, 95% CI 0.11 to 0.71, I^2^ = 58%) and leukemia (SMD = 1.74, 95% CI 0.92–2.56, I^2^ = N/A) (Fig. 3). Figure 3 shows the funnel plot of included studies.

#### Comparison 2 exercise capacity

**Fig. 4:**
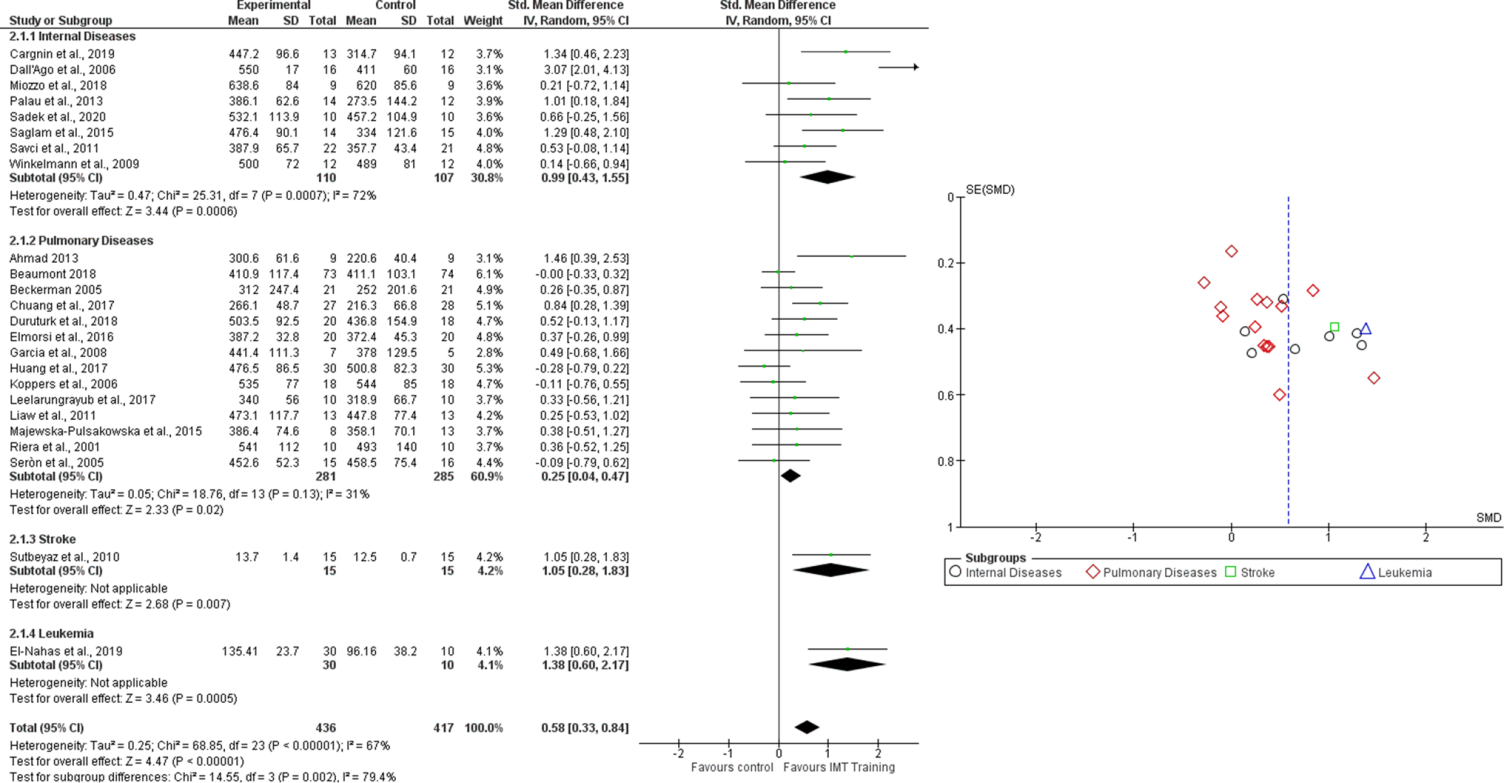
Comparison 2. RMT versus other interventions. Outcome: 2.1 Exercise Capacity, likewise Funnel plot of Comparison 2. RMT versus other interventions. Outcome: 2.1 Exercise Capacity

A total of 24 studies were included and divided in four subgroups (i.e. internal diseases, pulmonary diseases, stroke and leukemia). The overall number of participants was 853 and analyses were performed with SMD and random effects model. Results showed a significant improvement in exercise capacity in participants who underwent RMT (SMD = 0.58, 95% CI 0.33–0.84, I^2^ = 67%). The same results were obtained for internal diseases (SMD = 0.99, 95% CI 0.43–1.55, I^2^ = 72%), pulmonary diseases (SMD = 0.25, 95% CI 0.04–0.47, I^2^ = 31%), stroke (SMD = 1.05, 95% CI 0.28–1.83, I^2^ = N/A) and for leukemia patients (SMD = 1.38, 95% CI 0.60–2.17, I^2^ = N/A) (Fig. 4). Figure 4 shows the funnel plot of the Comparison 2.

#### Comparison 3 respiratory function–FEV1

**Fig. 5:**
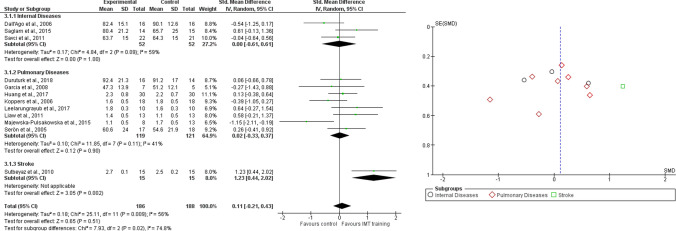
Comparison 3. RMT versus other interventions. Outcome: 3.1 Respiratory Function—FEV1, likewise Funnel plot of Comparison 3. RMT versus other interventions. Outcome: 3.1 Respiratory Function—FEV1

Twelve studies with 374 participants were analyzed. Analyses were performed with SMD, as studies assessed respiratory function by using FEV1 values but with different unit of measure. No significant difference was found between the two treatments (SMD = 0.11, 95% CI − 0.21–0.43, I^2^ = 56%), as well as for the subgroups internal diseases (SMD = 0.00, 95% CI − 0.61–0.61, I^2^ = 59%) and pulmonary diseases (SMD = 0.02, 95% CI − 0.33–0.37, I^2^ = 41%). A significant improvement in respiratory function assessed with FEV1 was found in stroke patients (SMD = 1.23, 95% CI 0.44–2.02, I^2^ = N/A) (Fig. 5). Figure 5 shows the funnel plot of the Comparison 3.

#### Comparison 4 respiratory function—MIP

**Fig. 6:**
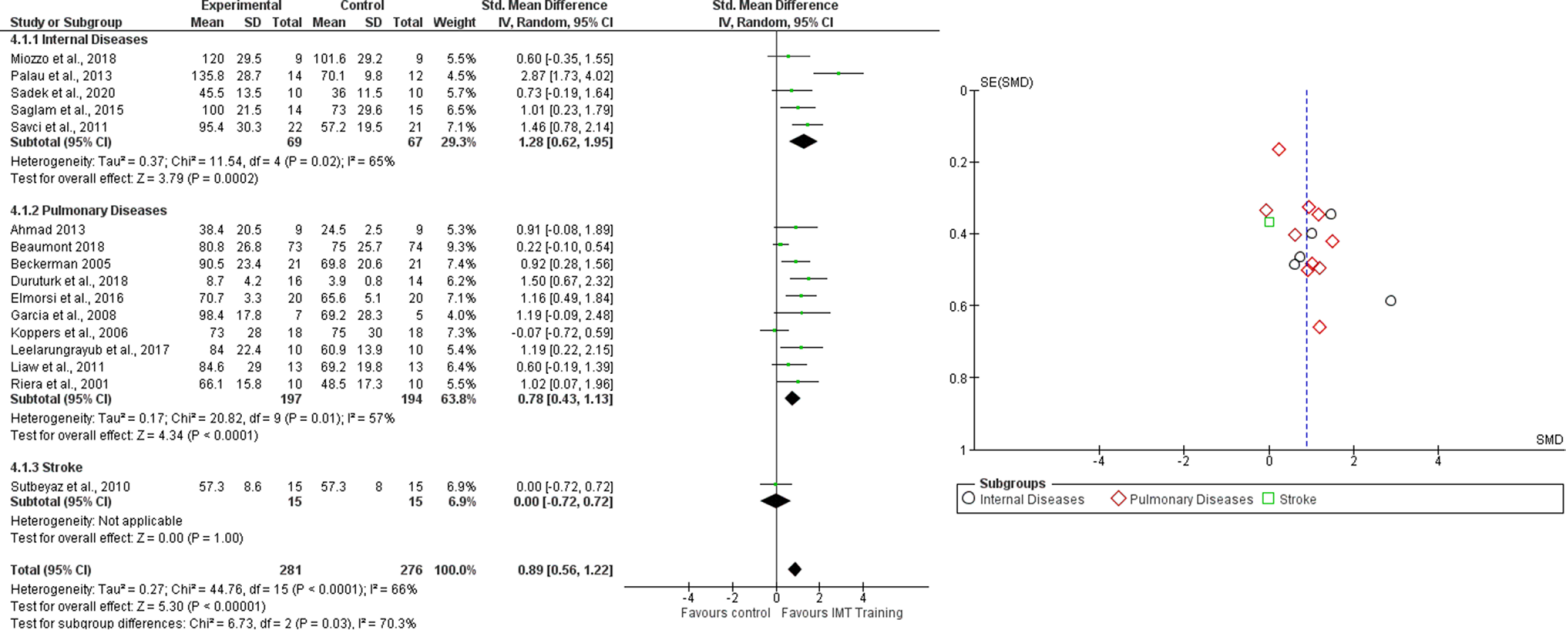
Comparison 4. RMT versus other interventions. Outcome: 4.1 Respiratory Function—MIP, likewise Funnel plot of Comparison 4. RMT versus other interventions. Outcome: 4.1 Respiratory Function—MIP

A total of 16 studies with 557 participants were included in the analysis, which were performed with SMD with random effects model. RMT resulted to be more effective than control condition (SMD = 0.89, 95% CI 0.56–1.22, I^2^ = 66%). Significant differences can be found in both internal diseases (SMD = 1.28, 95% CI 0.62–1.95, I^2^ = 65%) and pulmonary diseases subgroups (SMD = 0.78, 95% CI 0.43–1.13, I^2^ = 57%), but not in stroke subgroup (SMD = 0.00, 95% CI − 0.72–0.72, I^2^ = N/A) (Fig. 6). Figure 6 shows the funnel plot of the included studies.

#### Comparison 5 respiratory function—FVC

**Fig. 7:**
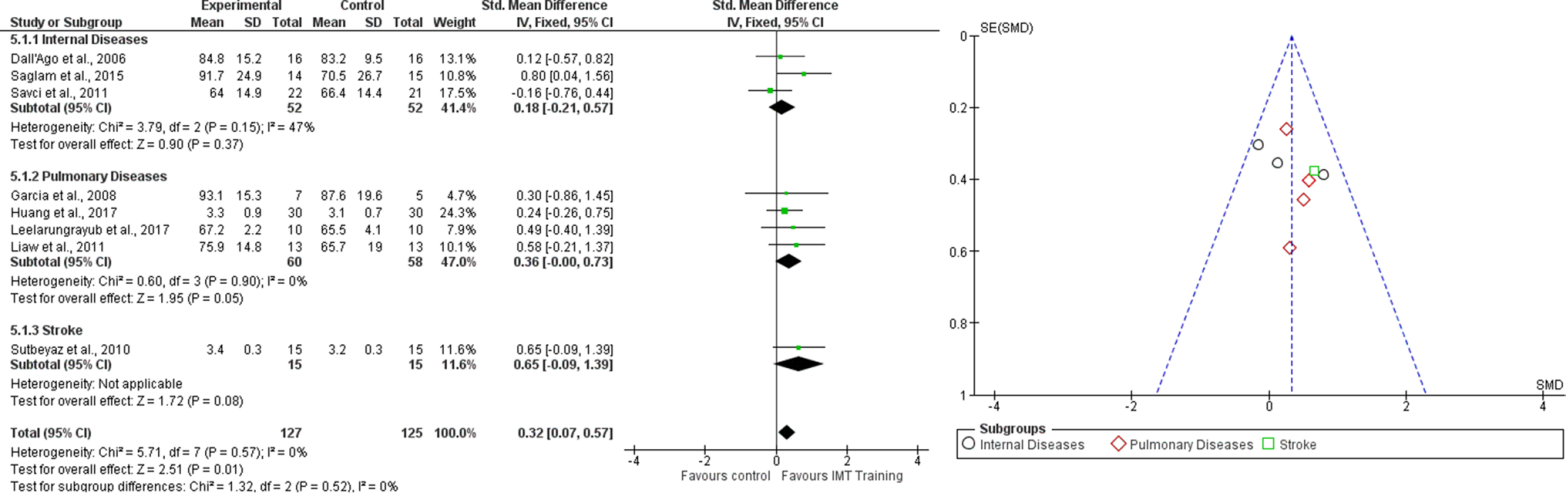
Comparison 5, RMT versus other interventions. Outcome: 5.1 Respiratory Function—FVC, likewise Funnel plot of Comparison 5, RMT versus other interventions. Outcome: 5.1 Respiratory Function—FVC

Eight studies and 252 participants were included. Analyses were performed using SMD with fixed effect model. Final result showed a significant difference between RMT and control condition (SMD = 0.32, 95% CI 0.07–0.57, I^2^ = 0%), but subgroup analyses revealed no difference between interventions for internal diseases (SMD = 0.18, 95% CI − 0.21–0.57, I^2^ = 47%), pulmonary diseases (SMD = 0.36, 95% CI − 0.00–0.73, I^2^ = 0%) and for stroke patients (SMD = 0.65, 95% CI − 0.09–1.39, I^2^ = N/A) (Fig. 7). Figure 7 shows the funnel plot of the included studies.

## Discussion

This review aimed to evaluate the effectiveness of RMT in internal and central nervous system disorders, and its effect on pulmonary function, exercise capacity and quality of life. Results suggest that patients with internal and central nervous system disorders who underwent RMT had better quality of life and improved significantly their performance in exercise capacity and in respiratory function assessed with FVC and MIP when compared to control conditions (i.e. no intervention, sham training, placebo or conventional treatments). No difference was found in respiratory function assessed with FEV1. However, results need to be considered carefully because of the following limitations.

In this systematic review, grey literature was not searched, in order to be as specific as possible, given also the large quantity of records obtained from the search in the primary databases. Furthermore, most of the analyses showed moderate-to-high values of heterogeneity, highlighting the presence of important inconsistency. The *Cochrane Handbook for Systematic Reviews of Interventions* suggests consideration of several possible sources of heterogeneity and asymmetry in funnel plots [29]. Indeed, the likelihood of drawing correct inferences from a meta-analysis decreases with increasing heterogeneity [62], thus investigation and plausible explanations for the presence of heterogeneity need to be carried out.

Results on quality of life, exercise capacity and respiratory function (FEV1 and MIP), are affected by clinical heterogeneity, likely due to difference in type and doses of intervention and comparator. In relation to the dose of therapy provided, this ranged from a total of 20 sessions over 10 days of treatment, to a maximum of 288 sessions over 1 year, ranging from twice a day all week to once a day twice a week. Likewise, in terms of training intensity, differences were present among the included studies. Respiratory resistance of the subjects ranged between 15 and 60% of the MIP. The included studies also varied in the devices used, including i.e. POWERbreathe, Threshold IMT, Respifit-S. This result tells us that a lack of agreement exists on the standard and/or optimal dose of therapy to deliver for achieving significant improvement, to patients with internal and central nervous system disorders. Furthermore, after analyzing the funnel plot of the Comparison 1, we observed a lack of studies in favour of RMT, suggesting the possibility of publication bias underestimating the effect of the experimental treatment, within the pulmonary diseases subgroup.

In Comparison 3 (Respiratory function—FEV1) selective outcome reporting affected the final results. In depth, we found that studies with unclear or high risk of bias for missing outcome data were less precise, underestimating significantly the effect of RMT on FEV1. Excluding these articles from the meta-analyses, results changed significantly in favour of RMT. This lack of methodology in trials conducting and reporting represents a major flaw in current literature in the field. Thus, it is essential that future trials reports will adhere to acknowledged standards, such as the CONSORT statement [63]. Furthermore, an evidence of imprecision in the effect estimate is observed, especially in Comparison 3. Most of the included studies had similar effects both in favour of the experimental or control treatment, making it difficult to draw a firm conclusion on the effectiveness of RMT.

The RMT resulted to be advantageous especially for the improvement of respiratory function assessed with FVC. In this case, the absence of heterogeneity and the significant results in favour of RMT provide evidence in favour of such training for patients with pulmonary dysfunction, nevertheless better training programs with standard dose of therapy need to be implemented, together with better trial reporting.

Comparing our results to previous research, it was indicated that RMT is effective to reduce postoperative pulmonary complications and length of hospital stay in patients undergoing surgery [64, 65]. Thus, the enhancement in the post-hospitalization phase of rehabilitation with RMT seems to bring additional benefits conveying to physical performance and quality of life. However, in line with the previous meta-analysis, the results also suggested no significant effect of RMT on FEV1 and the marginal on FVC [66]. Thus, it seems likely that elastic recoil and lung tissue properties, rather than expiratory muscle strength, may determine the maximum expiratory flow. This, in turn, is related to the pathomechanism of respiratory muscle weakness caused by lung tissue changes, or weakness due to skeletal muscle weakness. It is assumed that combined expiratory and inspiratory training may have a greater effect on lung function by increasing inspiratory reserve volume and elastic recoil and yielding significant improvements in FEV1 [14]. Therefore, it might be presumed that the patients had better perception of improvements at the level of activity, participation and quality of life, rather than structure and function (ventilator) aspects of the lung. Moreover, the novelty of this meta-analysis lies in the summarization of the effectiveness of RMT in several health areas in different patient groups. To our knowledge, a review with different disease classifications has not been performed previously. This approach was driven by an attempt to estimate the results obtained to the population of patients after hospitalization for COVID-19.

One should also consider the results obtained in the context of the current pandemic situation, with the first published clinical trials indicating the effectiveness of RMT on pulmonary functions, dyspnea, functional performance and QOL, after weaning from mechanical ventilation [67]. Although the role of RMT in mitigating the respiratory complications of viral infection has not yet been confirmed, the available evidence seems to suggest that this training may help reduce the risk of serious complications during viral infection [68]. It is also worth noting that RMT can be performed independently by the patient at home [69]. This opens many possibilities for the implementation of telerehabilitation or using new technologies [70]. Our team is currently running an ERS-funded project under Long-Term Research Fellowships. This project produced software to perform RMT in virtual reality. Based on our previous observations, we hypothesize that the virtual environment will lead to stress reduction during therapy [71–73]. Although the clinical experiment is currently in the implementation phase, it seems that lowering stress levels in patients after COVID-19 hospitalization may be one of the pillars of comprehensive pulmonary rehabilitation.

## Conclusion

Respiratory muscle training seems to be more effective than control conditions (i.e. no intervention, sham training, placebo or conventional treatment), in patients with pulmonary dysfunction due to internal and central nervous system disorders, for quality of life, exercise capacity and respiratory function assessed with MIP and FVC, but not with FEV1. However, standardized training programs with optimal dose of therapy need to be developed, and better trial reporting need to be implemented.

## Data Availability

The data presented in this study are available on request from the corresponding author.
